# Case Report: Diagnostic challenges in cervical small cell neuroendocrine carcinoma of a reproductive-age woman

**DOI:** 10.3389/fonc.2025.1692412

**Published:** 2025-11-10

**Authors:** Yitong Du, Huiling Yang, Yichen Feng, Zejun Wu, Mengyao Li, Tingting Zhu, Yali Zhuang

**Affiliations:** 1 Anhui Medical University, Hefei, China; 2 Department of Obstetrics and Gynecology, Maternal and Child Medical Center of Anhui Medical University, Hefei, China

**Keywords:** case report, small cell neuroendocrine carcinoma, uterine cervix, diagnosis, therapy, prognostic factors

## Abstract

Small cell neuroendocrine carcinoma of the cervix (SCNEC) is a rare and highly aggressive malignancy with a poor prognosis. We report the case of a 36-year-old nulliparous woman with a history of HPV-18 infection and repeated cytology showing atypical squamous cells of undetermined significance (ASC-US), while multiple biopsies remained negative. She initially underwent surgery for multiple uterine leiomyomas and severe endometriosis. Ten months later, she presented with acute pelvic pain, rectal pressure, and minor vaginal bleeding. Combined hysteroscopy and laparoscopy revealed suspicious lesions, and histopathology with immunohistochemistry confirmed International Federation of Gynecology and Obstetrics (FIGO) stage IVB SCNEC. Postoperatively, she received platinum-based chemotherapy followed by salvage immunotherapy. Despite a transient decline in tumor markers, the disease progressed rapidly, with enlargement and confluence of pelvic lesions and extensive bone metastases, ultimately resulting in death from multiorgan failure. This case highlights the diagnostic challenges, early systemic dissemination, and poor outcomes of SCNEC, underscoring the urgent need for more effective therapeutic strategies.

## 1 Introduction

Neuroendocrine carcinoma of the cervix (NECC) is a rare and highly aggressive cervical malignancy, accounting for approximately 1.4% of all invasive cervical cancers (1).According to the World Health Organization (WHO) classification, NECC can be divided into low-grade tumors (typical carcinoid and atypical carcinoid) and high-grade tumors (small-cell carcinoma and large-cell carcinoma). Among these, small-cell neuroendocrine carcinoma (SCNEC) is the most common subtype, representing about 80% of cases (1), with a mean age at diagnosis of 48.1 years (2). Even when clinically confined to the cervix, NECC demonstrates a marked propensity for early lymphatic and hematogenous dissemination and carries a high risk of lymph node metastasis (1). Early recognition is therefore crucial for improving the prognosis of patients with SCNEC, as it enables timely intervention that may delay disease progression and improve survival. Here, we present the case of a 36-year-old woman with SCNEC and summarize its clinical features, histopathology, treatment, and outcome.

## 2 Case report

**Figure 1:**
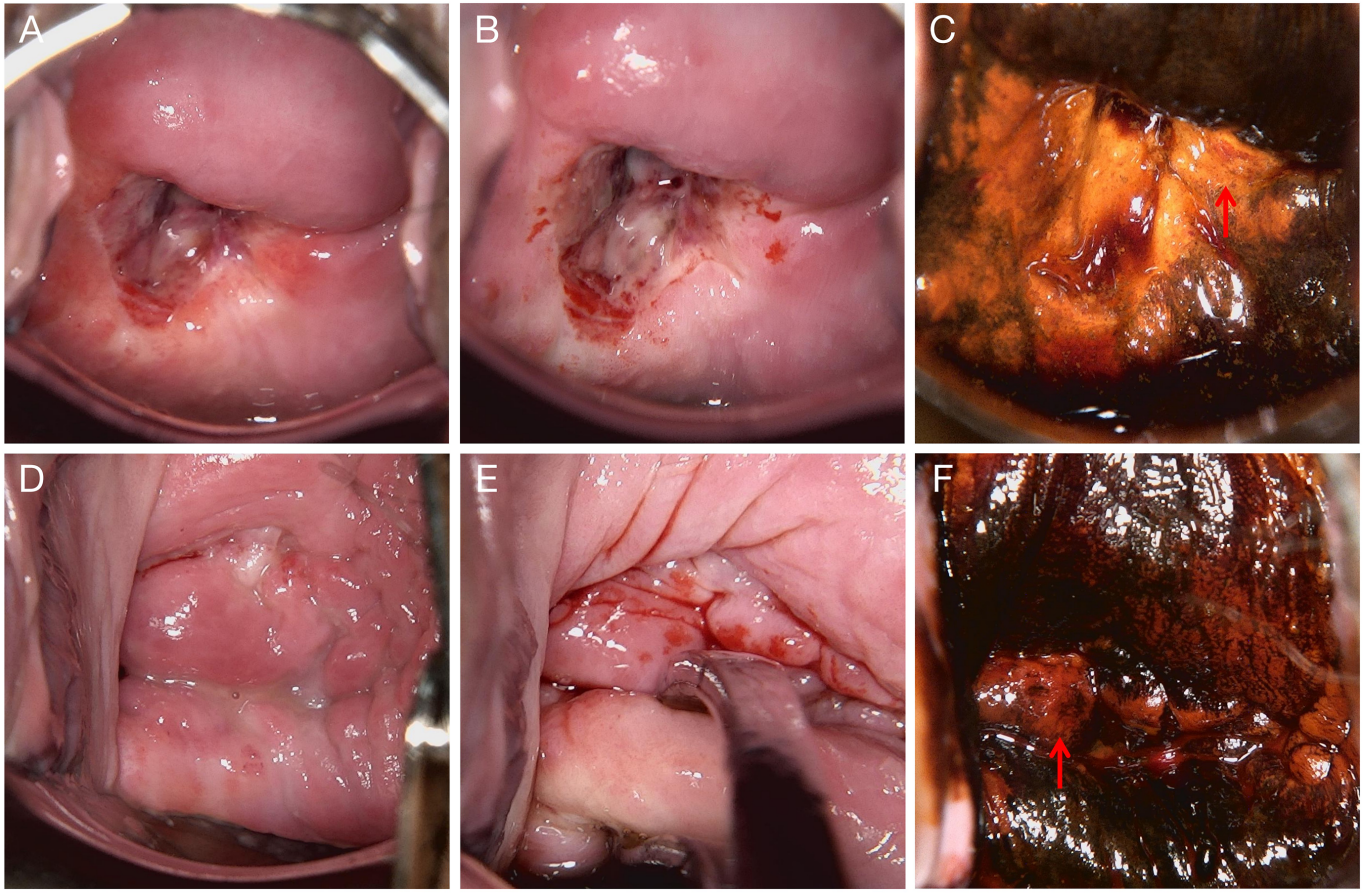
Colposcopic findings from two examinations. **(A–C)** Examination performed on March 8, 2022. **(A)** Native cervical appearance. **(B)** Acetowhite test showing dense acetowhite epithelium. **(C)** Lugol’s iodine staining demonstrating iodine-negative (unstained) areas suggestive of abnormal epithelium, with biopsy taken from the 3 o’clock position. **(D–F)** Examination performed on September 16, 2022. **(D)** Native cervical appearance. **(E)** Acetowhite test showing faint acetowhite changes. **(F)** Lugol’s iodine staining demonstrating iodine-negative (unstained) areas suggestive of abnormal epithelium, with biopsy taken from the 9 o’clock position. The biopsy areas for **(A–C)** and **(D–F)** are indicated with red arrows.

We present the case of a 36-year-old woman diagnosed with small cell neuroendocrine carcinoma of the cervix (SCNEC), a rare and highly aggressive malignancy. She was gravida 0, para 0, remarried, and had a history of cigarette smoking, alcohol consumption, and syphilis. The patient was first admitted to our hospital in March 2022 with a 6-month history of progressively worsening dysmenorrhea. Gynecological examination revealed abnormal cervical morphology, characterized by a hollow, firm cervix with contact bleeding. The uterus was anteverted and enlarged to the size of a 2-month pregnancy, with a palpable mass in the left adnexal region. Serum cancer antigen 125 (CA125) was elevated at 160.1 U/mL, while other tumor markers remained within normal limits. ThinPrep cytologic test (TCT) revealed atypical squamous cells of undetermined significance (ASC-US), and high-risk human papillomavirus (HPV) testing was positive for type 18. Colposcopy with acetic acid and Lugol’s iodine staining showed suspicious areas; however, a directed cervical biopsy was obtained from the 3-o’clock position, together with endocervical curettage (ECC), which revealed only chronic inflammation without evidence of neoplasia (**Figures 1A–C**).

Transvaginal ultrasound demonstrated multiple uterine fibroids and a 4.3 × 4.3 cm hypoechoic mass with internal echogenic foci in the left ovary. A 7 × 1 mm hyperechoic linear structure within the cervix was also noted, suggestive of calcification. The patient subsequently underwent laparoscopic myomectomy, bilateral ovarian cystectomy, and cervical biopsy. Pathological examination confirmed uterine leiomyomas, endometriotic cysts, and pelvic adhesions, while the cervical biopsy again showed only chronic inflammation. Endometriosis was staged as IV according to the revised American Society for Reproductive Medicine (rASRM) classification system (total score: 74), with an Endometriosis Fertility Index (EFI) score of 4.

At the 6-month follow-up, the patient remained positive for HPV-18, and repeated cervical cytology consistently showed ASC-US. A repeat colposcopy in September 2022 demonstrated an irregular cervical surface, with a defect at the 9 o’clock position. Acetic acid test and Lugol’s iodine staining revealed the 6 o’clock position as a suspicious area, leading to directed biopsy and concurrent endocervical curettage (ECC) (**Figures 1D–F**). The patient subsequently completed six cycles of gonadotropin-releasing hormone agonist (GnRH-a) therapy. Menstruation resumed three months after cessation of treatment, with reduced flow and mild dysmenorrhea. However, follow-up ultrasonography at a local hospital showed a 57 × 30 mm anechoic lesion with incomplete septation in the right adnexal region. During the next menstrual cycle, the patient developed prolonged bleeding with approximately double her baseline flow and severe dysmenorrhea requiring analgesics. In January 2023, she experienced acute lower abdominal pain accompanied by a sensation of rectal pressure and scant bloody vaginal discharge.

**Figure 2:**
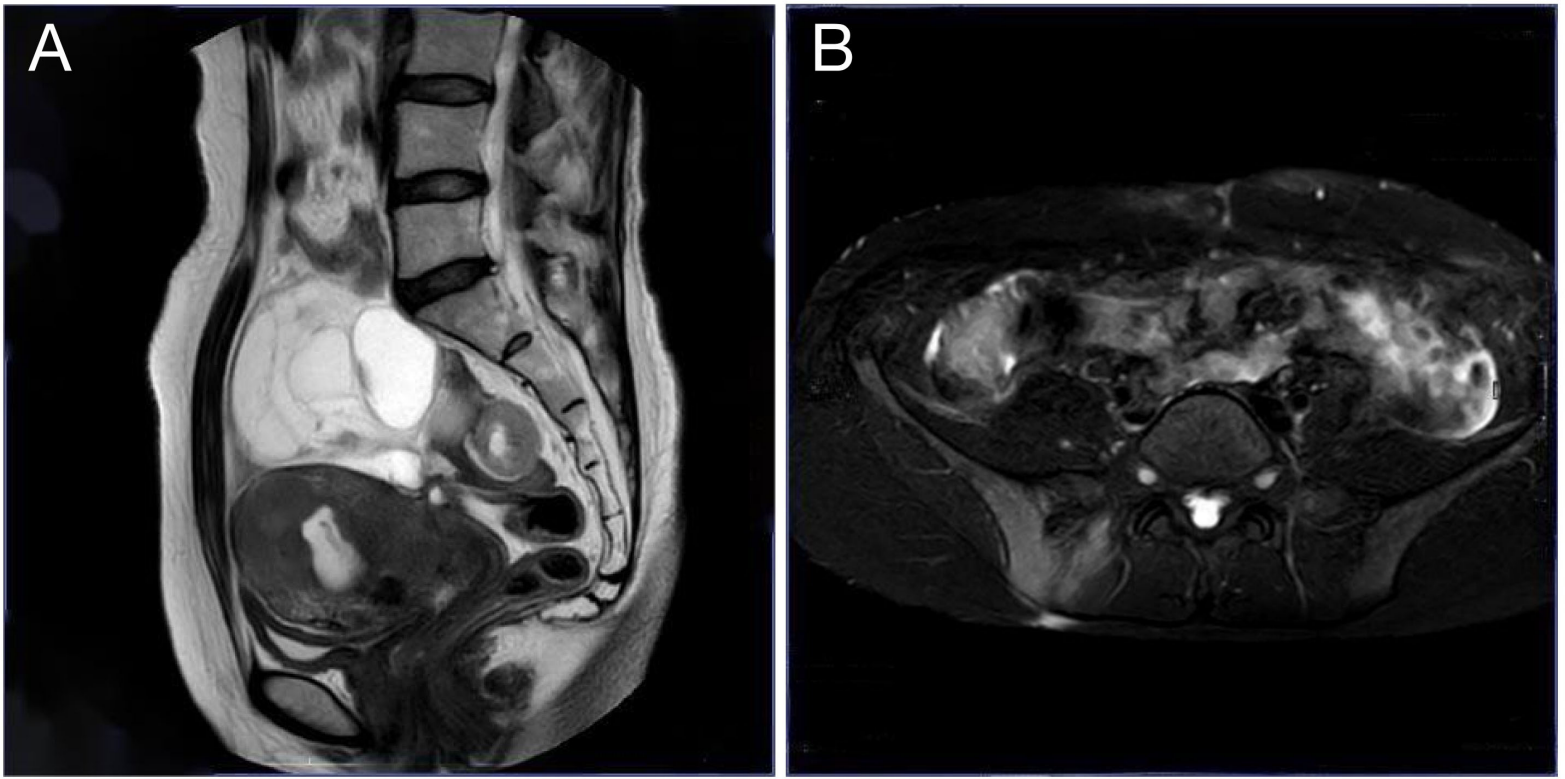
Pelvic MRI. **(A)** Sagittal T2-weighted image shows a cystic pelvic mass compressing the uterus and rectum; **(B)** Axial spectral attenuated inversion recovery (SPAIR) image highlights the mass with well-defined borders and involvement of the sacroiliac joints.

Upon readmission, pelvic examination revealed a markedly shortened, enlarged, irregularly shaped, and firm posterior cervical lip with restricted mobility and positive cervical motion tenderness. The uterus was enlarged to approximately 10-week size, firm, tender, and fixed. Bilateral adnexal masses were palpable (6 cm on the left, 10 cm on the right) with associated tenderness but no rebound or guarding. Laboratory testing revealed an anti-Müllerian hormone (AMH) level of 0.528 ng/mL, CA125 increased to 171.57 U/mL, human epididymis protein 4 (HE4) markedly elevated to 237.80 pmol/L, and carcinoembryonic antigen (CEA) mildly elevated at 5.14 ng/mL. Other tumor markers, including squamous cell carcinoma antigen (SCC), alpha-fetoprotein (AFP), carbohydrate antigen 15-3 (CA15-3), and carbohydrate antigen 19-9 (CA19-9), remained within normal ranges. Repeat TCT again indicated ASC-US. Imaging studies revealed a heterogeneous cervical echo pattern on transvaginal ultrasound, with an intrauterine separation measuring approximately 8 mm containing flocculent material. Two large adnexal masses with indistinct margins and internal vascularity were noted on either side of the uterus. In addition, hyperechoic hepatic lesions suggestive of hemangiomas were observed, along with polypoid changes in the gallbladder. Pelvic MRI demonstrated multiple ill-defined pelvic masses, abnormal signal changes in the right iliac crest and femoral head suggestive of malignancy, signal abnormalities in the uterus and cervix with hematometra, bilateral hydrosalpinges, as well as pelvic lymphadenopathy and effusion (**Figure 2**).

**Figure 3:**
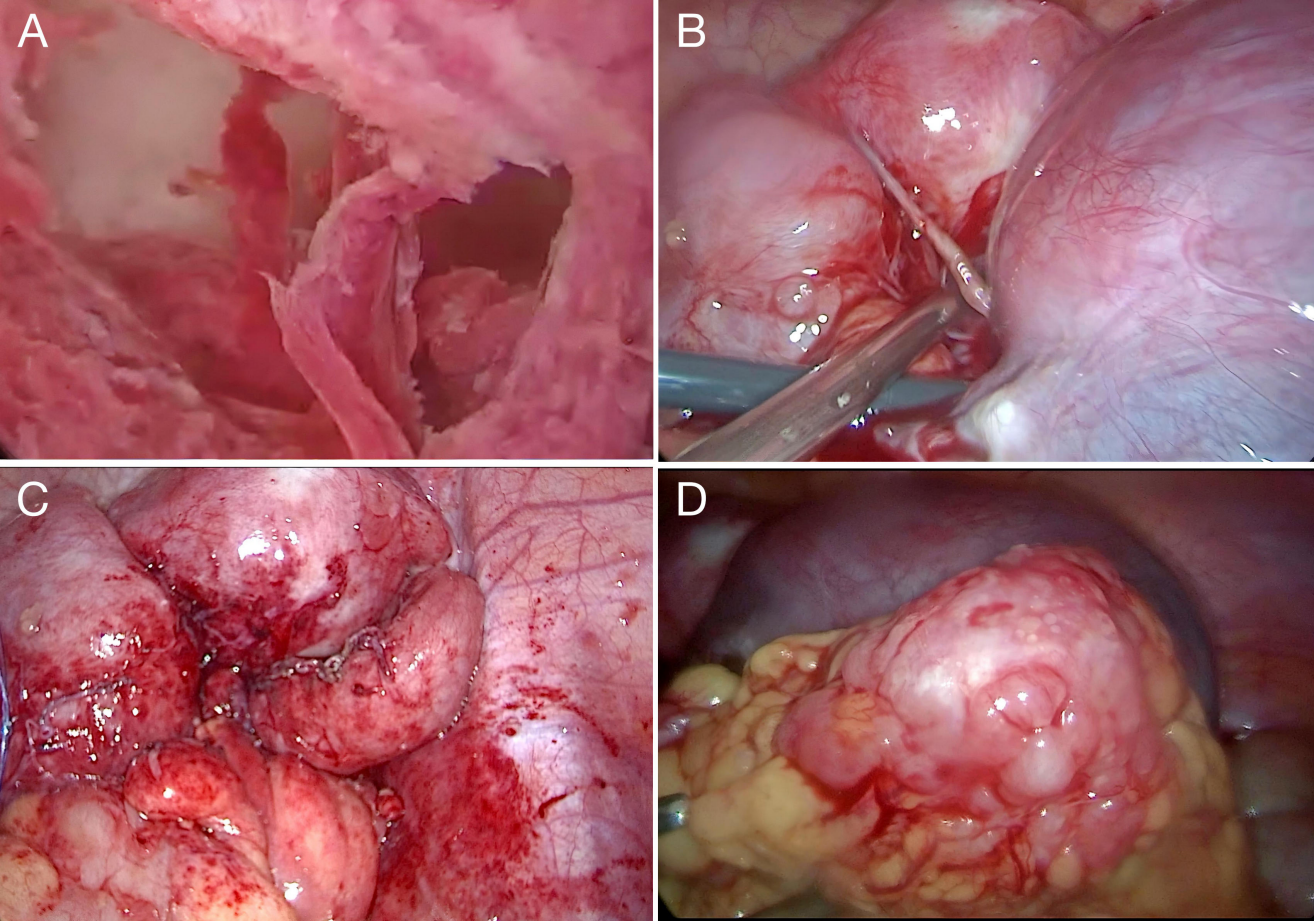
Operative findings. **(A)** Hysteroscopy revealed irregular thickening of the endometrium with hematometra. **(B)** Laparoscopy showed bilaterally enlarged ovaries, particularly on the right, adherent to the uterus and pelvic wall. **(C)** Thickened fallopian tubes fixed to the pelvic wall, with multiple peritoneal nodules. **(D)** The omentum was thickened with nodular and miliary lesions.

**Figure 4:**
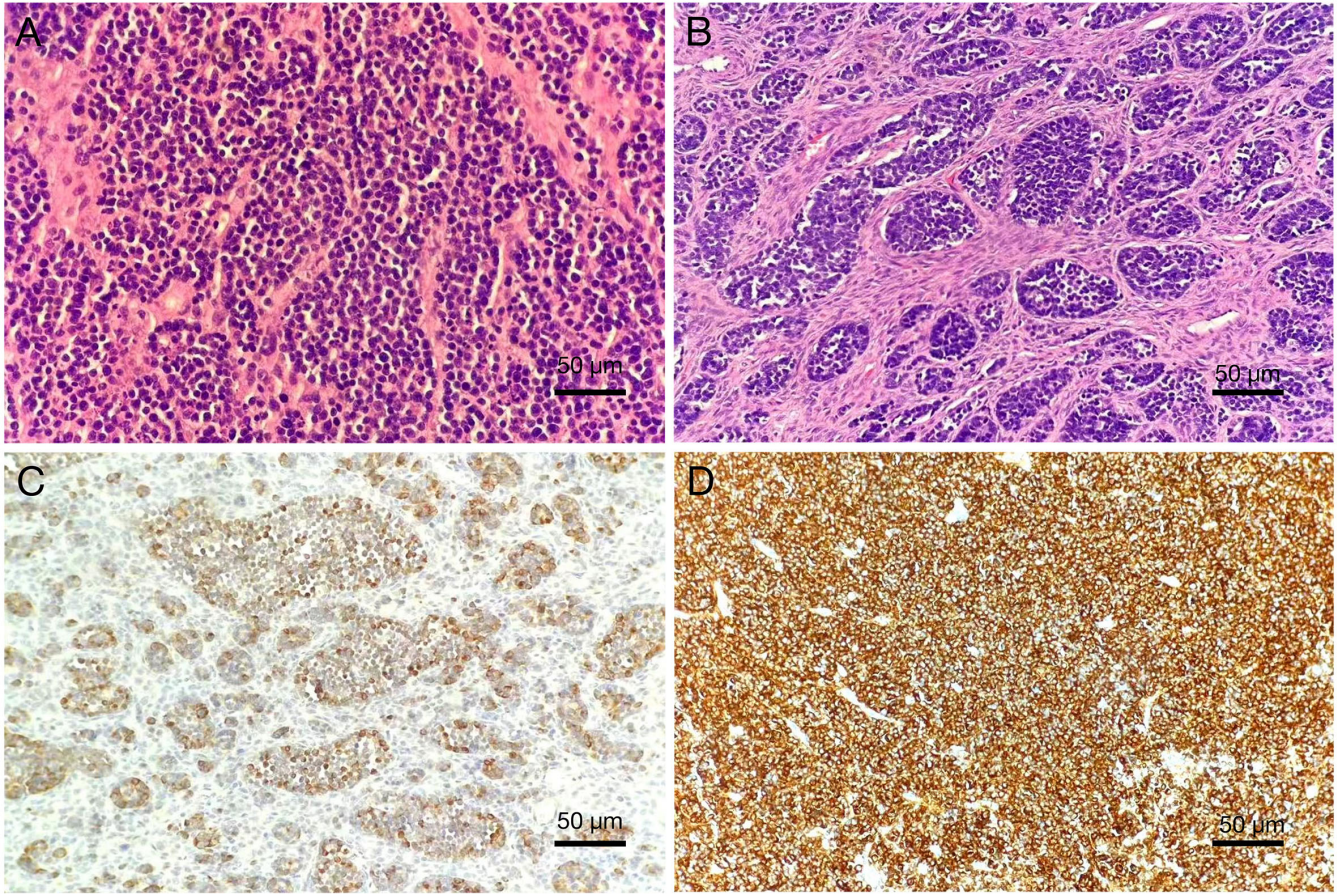
Pathological features of cervical small-cell neuroendocrine carcinoma (200×). **(A)** Hematoxylin and eosin (HE) staining shows small tumor cells with round morphology, arranged in sheets, with hyperchromatic nuclei, characteristic “salt-and-pepper” chromatin, scant cytoplasm, nuclear molding, and the presence of mitotic figures and apoptotic bodies. **(B)** HE staining demonstrates nests and trabeculae of tumor cells infiltrating the cervical stroma. Immunohistochemistry: Tumor cells are diffusely positive for **(C)** Chromogranin A (CgA) and **(D)** Synaptophysin (Syn), confirming neuroendocrine differentiation.

To further evaluate the pelvic masses, the patient underwent combined hysteroscopy and laparoscopy. The combined hysteroscopic–laparoscopic approach was performed primarily for diagnostic exploration, as malignancy had not yet been established preoperatively. Hysteroscopy revealed a firm, stenotic cervical canal and irregularly thickened endometrium with hematometra, from which biopsies of the endometrium and cervical os were obtained. Laparoscopy demonstrated approximately 100 mL of yellowish pelvic fluid, thickened omentum with nodular and miliary lesions, a 9 cm cystic mass in the right ovary adherent to the uterus and pelvic wall, and a 6 cm left ovary with a smooth surface. Both fallopian tubes were thickened and fixed. A retroperitoneal solid mass extended to the right pelvic wall with indistinct borders, and multiple nodules were observed along the right side of the rectum (**Figure 3**). The right ovary was subsequently excised, and ascitic fluid and omental tissue were collected for pathological evaluation. Final histopathology revealed a small-cell malignant tumor, and immunohistochemistry confirmed the diagnosis of small-cell neuroendocrine carcinoma, involving the submucosa of the cervical os, right ovary, and surface of the omental mass. Immunohistochemical staining was positive for cytokeratin AE1/AE3, epithelial membrane antigen (EMA), chromogranin A (CgA), synaptophysin (Syn), thyroid transcription factor-1 (TTF-1), CD99 (partial expression), Ki-67 (proliferation index 90%), cyclin-dependent kinase inhibitor 2A (p16), and SWI/SNF-related matrix-associated actin-dependent regulator of chromatin subfamily A member 4 (SMARCA4); weakly positive for paired box gene 8 (PAX-8); variably positive for tumor protein p53 (p53); and negative for leukocyte common antigen (LCA), p40, Wilms tumor 1 (WT-1), estrogen receptor (ER), and progesterone receptor (PR) (**Figure 4**). The final clinical diagnosis was International Federation of Gynecology and Obstetrics (FIGO) stage IVB SCNEC.

**Figure 5:**
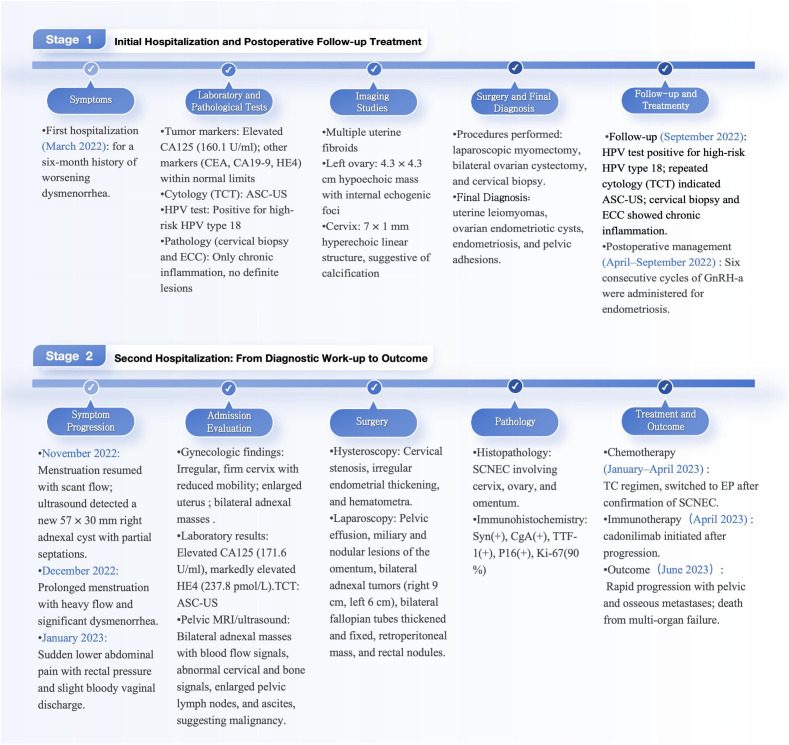
Timeline of the patient.

The patient underwent systemic chemotherapy, initially with carboplatin plus nab-paclitaxel (TC), later changed to cisplatin plus etoposide (EP) after confirmation of SCNEC. CA125 decreased, but HE4 remained elevated, suggesting ongoing tumor activity. Given the suboptimal response, cadonilimab, a bispecific antibody targeting programmed death-1 (PD-1) and cytotoxic T-lymphocyte-associated protein 4 (CTLA-4), was administered as salvage immunotherapy. Despite aggressive systemic therapy, imaging showed progressive disease. Pelvic MRI revealed multiple confluent tumor lesions with indistinct borders from surrounding bowel, and an 8.7 cm mass in the right sacroiliac joint and iliac bone. Extensive bony metastases were seen in the lumbar spine, sacrum, pubis, acetabulum, and proximal femur. Contrast-enhanced CT of the upper abdomen revealed suspicious metastatic foci in the spleen and L3 vertebral body. With rapid progression and poor performance status, she could not complete therapy and died of multiorgan failure. See (**Figure 5**) for a timeline of the patient’s clinical course.

## 3 Discussion

In developing countries, the incidence and mortality of cervical cancer remain disproportionately high due to the absence of systematic screening programs. While early detection and timely treatment significantly improve survival outcomes for most cervical cancer subtypes (3), SCNEC stands out as a rare and highly aggressive variant. Meta-analyses have shown that its 5-year survival rate is only 20%, compared with 74.3% for squamous cell carcinoma and 64.6% for adenocarcinoma (4). This remarkable disparity highlights the aggressive biological nature of SCNEC as well as the lack of robust, evidence-based therapeutic strategies, together posing major challenges for clinical management.

SCNEC most commonly presents with vaginal bleeding or a cervical mass as the initial symptom (5). A minority of patients may exhibit endocrine manifestations caused by multiple hormone abnormalities, including ectopic secretion of adrenocorticotropic hormone, antidiuretic hormone, and insulin (5–8). In this case, the patient instead developed acute lower abdominal pain following nonspecific menstrual abnormalities, an atypical presentation for cervical cancer. The patient had a prior history of stage IV endometriosis, which initially biased the clinical impression toward recurrence or complications of the pre-existing disease. Within this diagnostic expectation framework, early warning signs of SCNEC were obscured by the prior medical history, and its insidious symptomatology failed to attract adequate attention, creating a classic diagnostic pitfall. Moreover, the patient had persistent HPV-18 infection, and repeated cervical cytology (TCT) examinations consistently reported atypical squamous cells of undetermined significance (ASC-US). However, multiple colposcopy-guided cervical biopsies and endocervical curettage (ECC) only revealed chronic inflammation without evidence of neoplasia, failing to suggest features of neuroendocrine carcinoma.

This diagnostic challenge is not uncommon. A retrospective study from Severance Hospital reported that the diagnostic accuracy of Pap smears in 27 patients with SCNEC was only 22.2%, further suggesting that this disease carries a high risk of missed or delayed diagnosis, particularly at an early stage (9). Several factors may account for the repeatedly negative biopsy findings. First, SCNEC is an exceptionally rare cervical malignancy, and its low incidence has resulted in limited clinical recognition and diagnostic experience. Second, small-sample biopsies may fail to capture the tumor core, particularly when lesions arise from the endocervical canal or show diffuse infiltrative growth. Third, the rapid progression of SCNEC means that tumor morphology can undergo substantial changes within a short period, further complicating early detection. Against this background, molecular biomarkers represent a valuable adjunct for improving diagnostic accuracy. Prior studies have demonstrated that DNA methylation of tumor suppressor gene promoters is an early event in cervical carcinogenesis. When combined with p16 and Ki-67 immunohistochemistry, this approach allows better discrimination of transforming CIN lesions, prediction of regression or progression, and guidance for individualized management (10).In the present case, however, such molecular tests, including promoter DNA methylation analysis and p16/Ki-67 dual staining, were not performed due to diagnostic limitations at that time. Therefore, for patients with persistent high-risk HPV infection and repeatedly abnormal cytological findings, a more systematic diagnostic approach should be adopted on the basis of routine colposcopic evaluation. This should include expanding the sampling range when appropriate (such as endocervical curettage or diagnostic conization), performing multi-site or repeated biopsies, and incorporating molecular testing—such as promoter DNA methylation markers and p16/Ki-67 dual staining—to improve the detection of occult lesions like SCNEC and reduce the risk of missed diagnosis.

Minimally invasive surgery, with its advantages of reduced intraoperative blood loss, shorter hospitalization, and faster postoperative recovery, has become an important diagnostic and therapeutic approach in gynecologic oncology. While laparotomy may be preferable when malignancy is strongly suspected, in this case the diagnosis was uncertain (11). A minimally invasive hysteroscopic–laparoscopic approach was therefore adopted, taking into account the patient’s young age, nulliparous status, and fertility concerns. Conversion to laparotomy remained an option if intraoperative findings had strongly suggested malignancy. Furthermore, preoperative MRI indicated possible bone metastasis, consistent with FIGO stage IV disease, which further reduced the potential benefit of laparotomy.

Histologically, SCNEC is characterized by small tumor cells with round or spindle-shaped morphology, scant cytoplasm, coarse granular and hyperchromatic nuclear chromatin, inconspicuous nucleoli, and frequent mitotic figures, nuclear molding, and apoptotic bodies (12). Definitive diagnosis, however, relies on immunohistochemistry. Multiple studies have shown that CD56 positivity in SCNEC can reach 100% in some case series, while chromogranin A (CgA), synaptophysin (Syn), and neuron-specific enolase (NSE) are expressed in approximately 70%, 85%, and 80% of cases, respectively (13). Among these, synaptophysin and CD56 are considered the most sensitive markers for neuroendocrine differentiation, whereas CgA is noted for its higher specificity. Thyroid transcription factor-1 (TTF-1) positivity is also frequently observed (14). The emerging marker insulinoma-associated protein 1 (INSM1) has shown high specificity as well, with positive staining in more than 95% of cases (15). In addition, p16 overexpression can indicate HPV-related etiology. Kasuga et al. reported that 72% of SCNEC cases were associated with high-risk HPV infection (14% HPV16, 86% HPV18), and multiple studies have confirmed that p16 is almost universally and diffusely strongly positive in SCNEC (16), further underscoring the close relationship between persistent high-risk HPV infection and tumor development. Ki-67, as an important indicator of proliferative activity, is valuable not only for diagnosis but also for prognostic assessment. According to WHO recommendations, a proliferative index exceeding 20% is indicative of a high-proliferation phenotype (14, 16, 17). This case highlights several important diagnostic and biological aspects of SCNEC. Immunohistochemically, AE1/AE3 (+) and EMA (+) supported an epithelial origin, while CgA (+) and Syn (+) confirmed neuroendocrine differentiation. The concomitant expression of TTF-1 (+) and p16 (+) was in line with the characteristic immunophenotype of SCNEC. In addition, the extremely high Ki-67 proliferation index (90%) underscored the aggressive growth potential of this tumor. From a molecular perspective, accumulating evidence indicates that ovarian small-cell carcinomas, particularly the hypercalcemic type, almost universally carry deleterious SMARCA4 mutations, leading to their reclassification as malignant rhabdoid tumors within the same pathological spectrum (16–18). Similar alterations have also been documented in distinct subsets of lung, colorectal, bladder, and breast cancers (19). By contrast, SMARCA4 immunopositivity in cervical SCNEC remains uncommon. The present findings therefore raise the possibility that aberrant SMARCA4 expression may represent a shared oncogenic mechanism across high-grade small-cell neoplasms at different anatomic sites, although its precise significance in cervical SCNEC requires further clarification. At presentation, the lesion was confined to the cervix, yet the disease rapidly disseminated to the pelvis, bone, and lymph nodes, reaching FIGO stage IVB within a short interval, underscoring the hallmark aggressiveness of SCNEC.

Despite the highly aggressive nature of SCNEC, no standardized treatment guidelines have been established to date. In current clinical practice, a multimodal approach combining surgery, chemotherapy, and radiotherapy is generally adopted. Treatment strategies are often extrapolated from those for cervical cancer and other neuroendocrine carcinomas, such as small-cell lung cancer and Merkel cell carcinoma (18).The 2021 National Comprehensive Cancer Network (NCCN) guidelines included specific recommendations for SCNEC. For patients with early-stage localized disease, surgery combined with chemoradiation is recommended; for those with locally advanced disease, concurrent chemoradiation is the mainstay; and for patients with distant metastases, supportive systemic therapy is advised. The standard chemotherapy regimen typically consists of etoposide plus a platinum agent (16).

Immunotherapy is a novel therapeutic approach that harnesses the body’s immune system to recognize and eliminate tumor cells. Although it has been widely applied in advanced or recurrent cervical cancer, evidence specific to SCNEC remains scarce, mainly derived from small trials and individual case reports. Most high-grade neuroendocrine carcinomas of the cervix exhibit PD-L1 negativity and microsatellite stability (MSS), which may account for their limited response to PD-1/PD-L1 monotherapy. Nevertheless, isolated reports have shown clinical benefit from single-agent nivolumab even in PD-L1–negative SCNEC (20). Given the histopathologic similarity between SCNEC and small-cell lung cancer (SCLC), treatment strategies are often extrapolated from SCLC, where combined immune checkpoint blockade with nivolumab and ipilimumab has achieved an objective response rate (ORR) of approximately 20% (21). Although robust clinical data in cervical neuroendocrine carcinoma remain lacking, a case of complete remission reported by Paterniti et al. (22) suggests that dual immune checkpoint blockade may hold therapeutic potential in this rare and aggressive entity. Moreover, combining immunotherapy with ionizing radiation or DNA-damaging chemotherapy may enhance PD-L1 expression, thereby expanding the therapeutic applicability of immune checkpoint inhibitors in high-grade neuroendocrine tumors (20).

Cadonilimab (AK104) is a first-in-class bispecific monoclonal antibody targeting PD-1 and CTLA-4. In a pivotal phase II trial involving patients with previously treated recurrent or metastatic cervical cancer, cadonilimab achieved an ORR of 33.0%, including a complete response rate of 12.0%; notably, the ORR reached 43.8% among PD-L1–positive patients (23). Although its efficacy in SCNEC has not yet been established, this dual-target mechanism provides a rational basis for further exploration in this highly aggressive and treatment-refractory subtype. In the present case, given the patient’s rapid disease progression, multiple metastases, and poor response to standard chemotherapy, cadonilimab was administered as exploratory salvage therapy.

Meanwhile, advances in precision oncology have revealed recurrent somatic alterations in key signaling pathways in SCNEC, including RTK/RAS, PI3K–AKT–mTOR, p53, and MYC. Recent genomic studies further indicate that mutations in KRAS, PIK3CA, and homologous recombination repair (HRR) genes, as well as amplifications of IRS2 and SOX2, occur relatively frequently in SCNEC. These molecular aberrations provide new perspectives for understanding tumor biology and highlight potential therapeutic targets. Correspondingly, agents such as MEK inhibitors, PI3K/mTOR pathway inhibitors, and PARP inhibitors have been proposed as potential treatment options for selected patients (24–26). The absence of PD-L1 and genomic testing represents a limitation of this study; future clinical practice should actively incorporate such assessments to guide individualized therapy. With the ongoing development of targeted agents, immunotherapies, and next-generation HPV vaccines, the prognosis of SCNEC—a notoriously aggressive and treatment-refractory subtype of cervical cancer—holds the potential for substantial improvement (3).

Current evidence indicates that the most critical prognostic determinant for SCNEC is tumor stage (1). In addition, age (27, 28), tumor size (28, 29), smoking history (16), lymph node metastasis (28–31), and a pure small-cell histologic subtype have also been recognized as independent adverse prognostic factors (1). A large multicenter retrospective study conducted by the Japanese Society of Gynecologic Oncology, which included 822 patients with SCNEC, demonstrated that adjuvant chemotherapy following surgery significantly improved survival compared with adjuvant radiotherapy or concurrent chemoradiation, particularly in T1bN1M0 disease (29). Furthermore, a multicenter retrospective study of 93 patients with stage I–II high-grade neuroendocrine carcinoma (HGNEC) revealed that lymphovascular space invasion and pelvic lymph node metastasis were both significant predictors of shorter disease-free survival, with the former also associated with poorer overall survival, underscoring the prognostic relevance of local invasion and regional spread (31). In the present case, the patient exhibited several unfavorable prognostic features, including advanced stage, lymph node metastasis, and a pure small-cell histologic subtype, all of which portend a poor overall prognosis. Consistently, previous reports have estimated the 5-year cancer-specific survival rate of SCNEC to be approximately 36.6%, with a median overall survival ranging from 22 to 25 months (1).

## 4 Conclusion

This case highlights the multiple challenges associated with the diagnosis and management of SCNEC. Its early clinical manifestations are often obscured by coexisting benign gynecologic conditions, and traditional cytological screening methods may fail to detect the disease. Definitive diagnosis relies heavily on histopathological evaluation combined with immunohistochemical profiling. SCNEC is characterized by its aggressive biological behavior and high metastatic potential. Even when initially confined, the disease can rapidly disseminate to distant sites, and the overall prognosis remains poor. Currently, there is no standardized treatment protocol; multimodal therapeutic strategies often draw upon clinical experience from both cervical cancer and small-cell lung carcinoma. Emerging approaches involving immunotherapy and targeted therapies also warrant close attention. This case underscores the importance of maintaining a high index of suspicion for neuroendocrine carcinoma in patients with high-risk HPV infection and atypical clinical presentations. Early histological assessment and comprehensive immunohistochemical analysis using multiple markers are essential for prompt diagnosis and treatment planning.

## Data Availability

The original contributions presented in the study are included in the article/supplementary material. Further inquiries can be directed to the corresponding author.
